# PCSF: An R-package for network-based interpretation of high-throughput data

**DOI:** 10.1371/journal.pcbi.1005694

**Published:** 2017-07-31

**Authors:** Murodzhon Akhmedov, Amanda Kedaigle, Renan Escalante Chong, Roberto Montemanni, Francesco Bertoni, Ernest Fraenkel, Ivo Kwee

**Affiliations:** 1 Dalle Molle Institute for Artificial Intelligence Research (USI/SUPSI), Manno, Switzerland; 2 Institute of Oncology Research (IOR), Bellinzona, Switzerland; 3 Swiss Institute of Bioinformatics (SIB), Lausanne, Switzerland; 4 Department of Biological Engineering, MIT, Cambridge, Massachusetts, United States of America; Hebrew University of Jerusalem, ISRAEL

## Abstract

With the recent technological developments a vast amount of high-throughput data has been profiled to understand the mechanism of complex diseases. The current bioinformatics challenge is to interpret the data and underlying biology, where efficient algorithms for analyzing heterogeneous high-throughput data using biological networks are becoming increasingly valuable. In this paper, we propose a software package based on the Prize-collecting Steiner Forest graph optimization approach. The PCSF package performs fast and user-friendly network analysis of high-throughput data by mapping the data onto a biological networks such as protein-protein interaction, gene-gene interaction or any other correlation or coexpression based networks. Using the interaction networks as a template, it determines high-confidence subnetworks relevant to the data, which potentially leads to predictions of functional units. It also interactively visualizes the resulting subnetwork with functional enrichment analysis.

This is a *PLOS Computational Biology* Software paper.

## Introduction

A tremendous quantity of high-throughput data at multiple layers of cellular systems has been profiled to study biological responses to perturbations and diseases. The current challenge is to develop methods for effective analysis of these data to better interpret underlying biology and identify functional units. Network modeling approaches are some of the techniques lately used for analysis of biological networks [1, 2]. Recently, the Prize-collecting Steiner Forest (PCSF) algorithm has been applied to interaction networks to find a subnetwork enriched for input data, which corresponds to relevant subunits in the interactome of a cell [3, 4].

The PCSF problem has NP-hard characteristics, and it requires a great deal of effort to find exact solutions on larger biological networks. Recently, we have developed a fast heuristic for the PCSF [5], and conducted a detailed performance comparison with existing methods [6]. As an extension to this, we present an R package that performs fast and user-friendly analysis of high-throughput data using the interaction networks as a template, and interprets the biological landscape of interactome with respect to the data. More specifically, the PCSF package allows the user to i) upload the interactome and patient data ii) compute the PCSF subnetwork solution iii) perform functional analysis on resulting subnetwork iv) and interactively visualize the final subnetwork with functional enrichment information.

## Design and implementation

### The PCSF methodology

The PCSF is a well-known problem in graph theory. Given an undirected graph *G* = (*V*, *E*), where the vertices are labeled with prizes *p*_*v*_ and the edges are labeled with costs *c*_*e*_ > 0, the goal is to identify a subnetwork *G*′ = (*V*′, *E*′) with a forest structure. The target is to minimize the total edge costs in *E*′, the total node prizes left out of *V*′, and the number of trees in *G*′. This is equivalent to minimization of the following objective function:

$$\begin{array}{c} F\left(G^{\prime }\right)=\text{Minimize}\sum_{e\in E^{\prime }}c_{e}+\beta \cdot \sum_{v\notin V^{\prime }}p_{v}+w\cdot k \end{array}$$ (1)

where *k* is the number of trees in the forest, and it is regulated by parameter *w*. The parameter *β* is used to tune the prizes of nodes relative to edge costs.

Recently, we have applied PCSF to biological networks in the Forest module of the Omics Integrator software [4]. In biological networks such as protein-protein interaction (PPI) networks, every vertex represents a biomolecule, and every edge corresponds to the cellular interaction between two biomolecules. Edges of the network are given costs which correspond to confidence or frequency of that interaction. The vertices of the network are given prizes according to the measurements of differential expression, copy number, or number of mutation for that gene or protein. The set of vertices that are assigned a prize are referred to as *terminal* nodes. Non-terminal nodes, which were not observed in the experimental data, may appear in the solution and are called *Steiner* nodes. After scoring the interactome, the PCSF is used to detect a relevant subnetwork (forest). The PCSF aims to identify neighborhoods in interaction networks potentially belonging to the key dysregulated pathways of a disease or experiment.

In order to avoid a bias for the hub nodes of PPI networks to appear in solution of PCSF, we use the method introduced in Forest [4], which penalizes the prizes of nodes according to their degree in the PPI. Within the implementation, we use the parameter *μ* to fine-tune the penalties as:

$$\begin{array}{c} p_{v}^{\prime }=p_{v}-\mu *\text{degree}\left(v\right). \end{array}$$ (2)

The parameter *μ* also affects the total number of *Steiner* nodes in the solution. The higher the value of *μ*, the smaller the number of *Steiner* nodes in the subnetwork, and vice-versa. The recommended range of *μ* for biological networks is between 1e-4 and 5e-2 to fine-tune the *Steiner*/*terminal* node ratio in the subnetwork and average *Steiner*/*terminal* in-degree ratio of the corresponding nodes in the original template network.

### Implementation, dependencies, and installation

The software was implemented in R environment, and easily can be installed within the R terminal. As input, the package requires a template network such as protein-protein interaction, protein-metabolite interaction or any other correlation-based interaction network, and it maps differentially expressed genes/proteins/metabolites from the high-throughput data as vertex prizes into the template network. Then, it computes and returns high-scoring neighborhoods to identify functional modules in the interactome. Required parameters are: *β*—for tuning the vertex prizes, *ω*—for regulating the number of distinct components in the subnetwork, and *μ*—for hub penalization.

The package has the following R-package dependencies:

- BH and igraph—for efficient graph handling and calculations,
- httr, methods, org.Hs.eg.db, and topGO—for enrichment analysis,
- Rcpp—to employ C++ source code within R,
- visNetwork—for visualization.

The dependencies are automatically installed along with the PCSF package. For more details about the package dependencies and installation we refer the reader to see the supplementary documents.

### The software test

A software was successfully installed and tested in the following environments: Mac OS X (10.12.4) R 3.4.0, Ubuntu (16.04) R 3.2.3, Windows 7 R 3.4.1. We have analyzed over 100 biological network instances within the computational performance comparison, and illustrated our method in a biological application to interpret the phosphoproteomic data derived from H358 cells, a model of lung cancer. Few dozens of people have tested the package on installation, debugging, parameter setting, and own data. We provided the interaction network and the phosphoproteomic data from the lung cancer within the package. Any bugs, suggestions and request related to the package can be reported throughout its GitHub repository (https://github.com/IOR-Bioinformatics/PCSF).

## Results

### Computational performance comparison

In this section, we compare the computational performance of our method with the message passing (MSGP) [3] algorithm. The belief propagation has been used in similar biological application such as identification of unknown protein associations [3], prediction of hidden components in regulatory networks [7], and reconstruction of multiple dysregulated pathways [8]. As a template network, we used an integrated interactome of proteins [9] and metabolites [10], which composed of 36892 nodes and 1016411 edges. We employed the phosphoproteomic data from the Breast Cancer patients in [11]. A network instances are generated by mapping each patient’s differential phosphoproteomic data as *terminal* nodes onto template interactome. We tested the methods for *ω* = {1, 2}, and provided the average statistics of 10 runs to maintain a fair comparison baseline.

The comparison statistics of the methods are reported in Table 1. The performance of the MSGP [3] algorithm [3] and our method are provided under the MSGP and PCSF columns, respectively. The *μ* value was set to 0 for both methods to be comparable, which removes its impact on the objective function values. The solution qualities and running times of the approaches are displayed in the table. For these large network instances, the PCSF provides comparable quality solutions to the belief propagation algorithm for both values of *ω*. On other hand, the PCSF significantly outperformed the belief propagation in terms of running times. There is approximately ten times speed up on average, and it can be useful to analyze large biological networks in a reasonable time.

**Table 1:** The results of the methods for the Breast Cancer network instances generated using the phosphoproteomic data in [11]. The performance of the message passing algorithm [3] and the proposed method are respectively displayed under MSGP [3] and PCSF for *ω* = {1, 2}. The OBJ column reports the quality of the solutions (objective function values) obtained by the methods, and the running times of the algorithms are displayed under t(s) in seconds. The average statistics of 10 runs provided by both algorithms are reported for each instance.

		*ω* = 1				*ω* = 2			
		MSGP [3]		PCSF		MSGP [3]		PCSF	
Instance	Terminals	OBJ	t(s)	OBJ	t(s)	OBJ	t(s)	OBJ	t(s)
Basal-AN-A0AL	92	26.94	1920	27.18	101	27.94	2025	28.18	100
Basal-BH-A18Q	283	80.81	2188	81.31	291	82.89	1532	82.8	297
Her2-C8-A12Z	63	19.42	1422	19.51	71	20.42	1151	20.51	71
Her2-C8-A12L	75	25.07	1002	25.31	83	26.07	1134	26.31	83
Her2-A2-A0EQ	139	35.02	1583	35.33	147	36.02	1545	36.33	148
Her2-C8-A135	193	52.83	1762	53.19	199	53.85	1680	54.22	200
LumA-AO-A0JJ	168	50.2	1970	50.57	176	51.38	1729	51.83	175
LumA-A8-A08Z	174	48.55	1749	48.81	179	49.61	1904	49.87	181
LumA-BH-A0C1	201	52.42	2092	52.82	210	53.42	1992	53.82	207
LumB-AN-A0AJ	149	42.94	2220	43.32	155	43.96	1732	44.35	156
LumB-A7-A0CJ	158	45.36	2506	45.84	167	47.02	1476	47.52	170
LumB-AR-A1AV	190	56.47	1656	56.88	199	57.85	1506	58.26	198
LumB-AO-A03O	193	54.95	1878	55.22	205	55.87	1239	56.22	197
LumB-BH-A0DD	230	60.31	2297	60.65	235	61.37	2358	61.71	238
LumB-A2-A0T3	248	65.83	1793	66.05	261	66.91	2053	67.13	256
	*mean*	47.81	1869	48.13	179	48.97	1670	49.27	178
	*std*	16.26	377	16.32	62	16.43	354	16.42	62

For more information about the PCSF algorithm and rigorous performance comparisons, the interested readers are referred to [6].

### Biological application

In this section, we demonstrate the usage of the PCSF package on biological data, discuss the package functionalities and its output within the R environment. We analyze differential phosphoproteomic data derived from H358 cells, a model of lung cancer, that were stimulated with TGF-*β*. These data were previously published in [12], and were also used to demonstrate Omics Integrator [4]. We construct a template PPI network from the STRING database (version 13) [9] and apply some filtering steps [6]. A named vector containing the proteomic data and a data frame containing the filtered PPI is available within the package. The template network and data are loaded into R environment as follows:

> data(“STRING”)
> ppi <- construct_interactome(STRING)
> data(“Tgfb_phospho”)
> terminals <- Tgfb_phospho

The resulting PPI network consists of 17581 edges and 15405 nodes, out of which 58 terminal nodes correspond to differentially phosphorylated proteins. After loading the PPI network and assigning the terminal prizes, we use PCSF() to find high-confidence subnetworks by providing parameters *β*, *ω* and *μ* as it is discussed in Section. The dynamic and interactive output subnetwork can be plotted with plot() function.

> subnet <- PCSF(ppi, terminals, w = 2, b = 1, mu = 0.0005)
> plot(subnet)

Given that the edge weights and input data were often derived from high-throughput data and are necessarily noisy, it is recommended to test the robustness of the solution. One way to do this is to solve the PCSF several times while adding noise to edge costs, and combine all results in the final subnetwork. Edges and nodes are then given scores indicating how many times they appeared in the solutions with varying edge costs.

> subnet <- PCSF_rand(ppi, terminals, n = 10, r = 0.1, w = 2, b = 1, mu = 0.0005)

Next, an enrichment analysis of the final subnetwork is performed for functional interpretation. The subnetwork is clustered using the edge betweenness clustering algorithm from the igraph R-package, and for each cluster, functional enrichment is done by employing either EnrichR [13] API or topGO [14] R-package that can be specified by the user. Note that EnrichR API requires a working internet connection to perform the enrichment. If not specified, the package defaults to EnrichR, it uses topGO if there is no internet connection.

**Fig 1:**
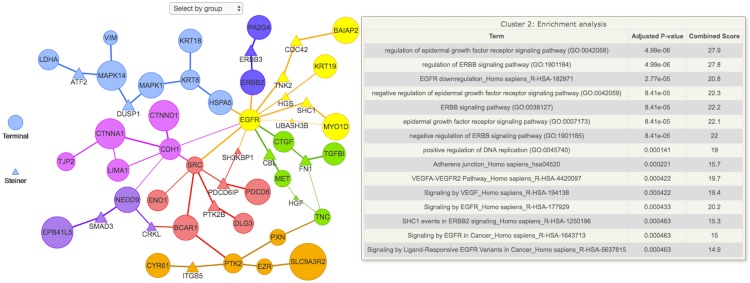
Functional enrichment analysis of the final subnetwork using the EnrichR API. The node sizes and edge widths are proportional to the amount of times that node or edge appeared in the noisy runs. Nodes are colored according to cluster membership. As in the EnrichR API, the p-value is calculated using the Fisher test and adjusted for multiple hypotheses. The top 15 functional enrichment terms for each cluster are ranked according to the adjusted p-value and displayed in a tabular format when the mouse hovers over a node in that cluster. Each cluster can be visualized separately by “Select by group” icon located at the top of the figure.

An interactive version of the annotated subnetwork can be visualized as in Fig 1. In the case of the Tgf-*β* stimulation data, the subnetwork provided by PCSF was enriched for relevant Gene Ontology terms like “mesenchymal-epithelial cell signaling” and “EGFR downregulation”. We also see Steiner nodes such as CBL and ITGB5, which have been shown to be involved in several models of non-small cell lung cancers [15], like the H358 cells. Therefore, we see that the PSCF algorithm points out proteins and pathways that are highly relevant to the system under study.

> res <- enrichment_analysis(subnet)
> plot(res$subnet)

## Availability and future directions

The source code of the package and the tutorials are available on GitHub at https://github.com/IOR-Bioinformatics/PCSF. The package is accompanied with the building and installing documentation, a detailed vignette and reference manual to demonstrate the usage of its functions. We used the MIT open source software license for the package.

The PCSF is a general method and it also can be applied to interpret multi-omics data for functional analysis. We plan to integrate genomic, transcriptomic, proteomic and metabolomic high-throughput data by mapping them onto biological networks and solving the PCSF. Heterogeneous omics data integration is extremely challenging task. At the same time, it is crucial to have overall picture and understanding of cellular mechanism in deep, and develop effective therapeutic strategies for complex diseases. Currently, the bioinformatics community is still missing effective data integration techniques, and we believe our method will be one-step contribution for improving the available methods in the literature.

The last but not least, the proposed package is significantly faster compared to the previous network based PCSF methods, which can analyze large biological networks in a reasonable time.

## Supporting information

S1 File A vignette for the package. (HTML)

S2 File A reference manual of the package. (PDF)
