# Supplementary material for: PCSF: An R-package for network-based interpretation of high-throughput data
Source: PLoS Comput Biol. 2017 Jul 31;13(7):e1005694. doi: 10.1371/journal.pcbi.1005694 (PMC5552342; doi:10.1371/journal.pcbi.1005694)
Supplement: S1 File — (HTML) [file pcbi.1005694.s001.html]

# PCSF Package

#### *Murodzhon Akhmedov, Amanda Kedaigle, Renan Escalante, Roberto Montemanni, Francesco Bertoni, Ernest Fraenkel, Ivo Kwee*

#### *February 21, 2017*

#### Introduction

A tremendous amount of high-throughput data at multiple layers of cellular systems has been profiled to study biological responses to perturbations and disease. The current challenge is to develop methods for effective analysis of these data to better interpret underlying biology and identify functional units. Network modeling approaches are some of the techniques lately used for analysis of biological networks (Dittrich et al. 2008). Recently, the Prize-collecting Steiner Forest (PCSF) algorithm has been applied to interaction networks to find a subnetwork enriched for input data, which corresponds to relevant subunits in the interactome of a cell (Bailly-Bechet et al. 2011, Tuncbag et al. (2016)).

The PCSF problem has NP-hard characteristics, and requires a great deal of effort to find exact solutions on larger biological networks. Recently, we have developed a fast heuristic for the PCSF (Akhmedov, Kwee, and Roberto 2016), and conducted a detailed performance comparison with existing methods (Akhmedov et al. 2017). As an extension to this, we present an R package that performs fast and user-friendly analysis of high-throughput data using the interaction networks as a template, and interprets the biological landscape of interactome with respect to the data. More specifically, the PCSF package allows the user to i) upload the interactome and patient data ii) compute the PCSF subnetwork solution iii) perform functional analysis on resulting subnetwork iv) and interactively visualize the final subnetwork with functional enrichment information.

#### The PCSF: Optimization Approach on Biological Networks

The PCSF is a well-known problem in graph theory. Given an undirected graph \(G = (V, E)\), where the vertices are labeled with prizes \(p\_{v}\) and the edges are labeled with costs \(c\_{e} > 0\), the goal is to identify a subnetwork \(G' = (V', E')\) with a forest structure. The target is to minimize the total edge costs in \(E'\), the total node prizes left out of \(V'\), and the number of trees in \(G'\). This is equivalent to minimization of the following objective function:

\[F(G')= \text{Minimize} \sum\_{e{\in}E'} c\_{e} + \beta\*\sum\_{v{\not\in}V'} p\_{v} + w\*k \label{GW}\]

where \(k\) is the number of trees in the forest, and it is regulated by parameter \(w\). The parameter \(\beta\) is used to tune the prizes of nodes relative to edge costs.

Recently, we have applied PCSF to biological networks in the Forest module of the Omics Integrator software (Tuncbag et al. 2016). In biological networks such as Protein-Protein Interaction (PPI) networks, every vertex represents a biomolecule, and every edge corresponds to the cellular interaction between two biomolecules. Edges of the network are given costs, which correspond to confidence or frequency of that interaction. The vertices of the network are given prizes according to the measurements of differential expression, copy number, or number of mutation for that gene/protein. The set of vertices that are assigned a prize are referred to as `terminal` nodes. Non-terminal nodes, which were not observed in the experimental data, may appear in the solution and are called `Steiner` nodes. After scoring the interactome, the PCSF is used to detect a relevant subnetwork (forest). The PCSF aims to identify neighborhoods in interaction networks potentially belonging to the key dysregulated pathways of a disease or experiment.

In order to avoid a bias for the hub nodes of PPI networks to appear in solution of PCSF, we use the method introduced in Forest (Tuncbag et al. 2016), which penalizes the prizes of nodes according to their degree in the PPI. The parameter \(\mu\) controls the size of these penalties: \[p'\_{v} = p\_{v} - \mu\*degree(v)\] The parameter \(\mu\) also affects the total number of `Steiner` nodes in the solution. The higher the value of \(\mu\), the smaller the number of `Steiner` nodes in the subnetwork, and vice-versa. The recommended range of \(\mu\) for biological networks is between `1e-4` and `5e-2` to fine-tune the `Steiner`/`terminal` node ratio in the subnetwork and average `Steiner`/`terminal` in-degree ratio of the corresponding nodes in the original template network.

#### Package Dependencies and Installation

The software was implemented in R environment, and easily can be installed within the R terminal. As input, the package requires a template network such as protein-protein interaction, protein-metabolite interaction or any other correlation-based interaction network, and it maps differentially expressed genes/proteins/metabolites from the high-throughput data as vertex prizes into the template network. Then, it computes and returns high-scoring neighborhoods to identify functional modules in the interactome. Required parameters are: \(\beta\) - for tuning the vertex prizes, \(\omega\) - for regulating the number of distinct components in the subnetwork, and \(\mu\) - for hub penalization.

The package has the following R-package dependencies that are automatically installed along with the package:

- `BH` and `igraph` - for efficient graph handling and calculations,
- `httr`, `methods`, `org.Hs.eg.db`, and `topGO` - to perform enrichment analysis,
- `Rcpp` - to employ C++ source code within R,
- `visNetwork` - for visualization.

In order to compile the source, Windows users should install the `Rtools` package by the following link that installs GCC and CMake.

The PCSF package and its dependencies can be installed on Mac OS, Linux and Windows by running the following commands in the R console.

```
source("http://bioconductor.org/biocLite.R")
biocLite("BiocInstaller")
install.packages("devtools", dependencies=TRUE)
install_github("IOR-Bioinformatics/PCSF", repos=BiocInstaller::biocinstallRepos(),
               dependencies=TRUE, type="source", force=TRUE)
```

#### Application

In this simple example application, we demonstrate the functions of the PCSF package and its usage. In this study, we analyze differential phosphoproteomic data derived from H358 cells, a model of non-small cell lung cancer, which were stimulated with TGF-β. These data were previously published (Thomson et al. 2011), and are the same ones used to demonstrate Omics Integrator (Tuncbag et al. 2016, Figure 4). We used the PPI network from the STRING database (version 13) (Szklarczyk et al. 2011) as a template network, in which the network edges have a confidence score `s(e)` computed from experimental channels. The low confidence edges with `s(e) < 0.5` are removed from the interactome to increase the reliability of computational findings. We convert edge confidence into edge cost: `c(e) = max(0.01, 1-s(e))`. The mutation data and PPI network is available in the package. Let’s load the library and data into an R session.

```
library(PCSF)
```

```
data("STRING")
data("Tgfb_phospho")
```

Users can construct a PPI network by providing their own interactome. An interactome data for `construct_interactome()` must be a `data.frame` composed of three columns, where each row corresponds to an edge, in which the first element is a `head`, the second element is a `tail`, and the last element represents the `cost` of the edge.

```
ppi <- construct_interactome(STRING)
ppi
```

```
## IGRAPH UNW- 15405 175821 -- 
## + attr: name (v/c), weight (e/n)
## + edges (vertex names):
##  [1] RNF14--SMAD4         RNF14--SMAD1         RNF14--EIF2B5       
##  [4] RNF14--EIF2B2        RNF14--EIF2B1        RNF14--TAGLN        
##  [7] RNF14--UBE2D2        RNF14--UBE2D1        RNF14--SMURF1       
## [10] RNF14--SMURF2        RNF14--NCOA4         RNF14--EXOSC3       
## [13] RNF14--EXOSC5        RNF14--HNRNPA1       RNF14--UBE2U        
## [16] RNF14--UBE2W         RNF14--TNFAIP3       RNF14--DKFZp586J0119
## [19] RNF14--ESR1          RNF14--UBC           RNF14--AR           
## [22] RNF14--UBE2V1        RNF14--IST1          RNF14--VDR          
## + ... omitted several edges
```

We then generate a vector named `terminals` that is composed of genes with prizes to be analyzed in the PCSF context. Here, our prizes come from the phosphoproteomic data derived from H358 cells. There are 58 proteins which were determined to be significantly modulated in phosphorylation after TGF-β stimulation, and the prize for each protein is the absolute value of the log fold change in phosphotyrosine abundance.

Users can generate this vector by providing their own data. It must be a named numeric vector, where the genes names are the same as in the interaction network and numeric values correspond to the importance of the gene within the study.

```
terminals <- Tgfb_phospho
str(terminals)
```

```
##  Named num [1:58] 0.81 0.29 2.68 1.54 0.43 0.84 0.84 0.76 0.88 0.96 ...
##  - attr(*, "names")= chr [1:58] "BAIAP2" "BCAR1" "CALM1" "CDH1" ...
```

After properly obtaining the PPI network and scoring it, we employ the PCSF approach to identify the subnetwork with the following function, in which the arguments are: `ppi` - a weighted PPI network, `terminals` - a named numeric vector which corresponds to the list of genes with prizes, `w` - a parameter for tuning the number of trees in the subnetwork, `b` - a numeric value for tuning the node prizes, `mu` - a parameter for hub penalization. This function outputs the subnetwork as an `igraph` object.

```
subnet <- PCSF(ppi, terminals, w = 2, b = 1, mu = 0.0005)
```

The dynamic and interactive version of final subnetwork can be plotted with the `plot.PCSF()` function, where it takes the arguments: `x` - the output obtained by PCSF approach, `style` - a boolean value to determine the visualization style of the network, `edge_width` - a variable to adjust edge widths, `node_size` - a numeric value to adjust the size of nodes, `node_label_cex` - a numeric value to set node label size, `Steiner_node_color` - a variable to color the `Steiner` nodes, `Terminal_node_color` - a variable to color `terminal` nodes. The node sizes and edge widths in the figure are proportional to the prize of nodes and probability of existence of the edges, respectively, and they are adjusted according to `node_size` and `edge_width` parameters.

```
plot(subnet, style = 1, edge_width=5, node_size=40, node_label_cex = 30, Steiner_node_color = "lightblue", 
            Terminal_node_color = "lightgreen")
```

Now, let’s observe how the hub penalization parameter is effecting the final subnetwork. We solve the PCSF with a higher `mu` value than previous one (`mu=0.05`), and plot the subnetwork. It is clear that when the `mu` value is increased, we get very limited number of `Steiner` nodes and a smaller subnetwork overall.

```
subnet <- PCSF(ppi, terminals, w = 2, b = 100, mu = 0.05)
plot(subnet, style = 1)
```

However, if we set `mu` to zero, we see that the forest relies on the hub node UBC, or the regulatory protein ubiquitin-C. These hub nodes are often not specific to the system in the experiment.

```
subnet <- PCSF(ppi, terminals, w = 2, b = 100, mu = 0)
plot(subnet, style = 1)
```

#### Adding random noise to the edge costs

We must be cautious making a biological interpretation of data based on a single run of PCSF. Since the PPI network is huge and data is noisy, it is possible to have some random nodes in the output forest of each run. In order to increase the robustness of the resulting structure, it is a reasonable approach to solve PCSF several times by adding some noise to edge costs each time, and combine all results in the final forest. The union of all outputs may explain the underlying biology better. Running the PCSF with noisy edge costs can be performed with the function below, where argument `n` determines the number of runs and argument `r` determines the level of noise.

```
subnet <- PCSF_rand(ppi, terminals, n=10, r = 0.1, w = 2, b = 1, mu = 0.0005)
```

#### Functional Enrichment Analysis

The enrichment analysis of the final subnetwork is performed with the `enrichment_analysis()` function. The subnetwork is clustered by edge betweenness algorithm from the `igraph` package, and for each cluster the functional enrichment is done by employing either EnrichR (Chen et al. 2013) API or topGO (Alexa and Rahnenfuhrer 2009) R-package that can be specified by the user. Note that EnrichR API requires a working internet connection to perform the enrichment. If not specified, the package defaults to EnrichR, it uses topGO if there is no internet connection.

The `enrichment_analysis()` fuction requires the following arguments: `subnet` - the final subnetwork obtained by the PCSF method, `mode` - a binary variable to choose the method for enrichment analysis, where 0 is for EnrichR API and 1 is for topGO package, and `gene_universe` - a complete list of genes (vector of gene symbols) used as background in enrichment analysis by topGO package.

It is required to define a `gene_universe` to use topGO, and the gene\_universe in this example is all the genes in the PPI template network. It performs enrichment analysis for `Biological_Process` terms in Gene Ontology database within our implementation.

```
library(topGO)
gene_universe <- V(ppi)$name
res <- enrichment_analysis(subnet, mode=1, gene_universe)
```

In contrast, EnrichR API does not require a `gene_universe`, and it gathers and combines the enrichment analyses for `GO_Biological_Process_2015`,`KEGG_2016`, `Reactome_2016`, and `BioCarta_2016` databases.

```
res <- enrichment_analysis(subnet)
```

The `enrichment_analysis()` returns an annotated subnetwork (`igraph` object) and a list of tables that contain full enrichments for each cluster. The subnetwork can be saved in a desirable format available in the `igraph` pacakge.

An interactive version of the annotated subnetwork is visualized by the `plot.PCSFe()` function, where each cluster is colored differently. The plotting function requires the following arguments: `x` - an annotated subnetwork obtained by the `enrichment_analysis()` function, `edge_weight` - a variable to set the edge weights, `node_size` - a variable to adjust the size of nodes, `node_label_cex` - a numeric value to adjust the node label size.

The node sizes and edge widths are proportional to the amount of times that node or edge appeared in the noisy runs. Nodes are colored according to cluster membership. As in the ENRICHR API, the p-value is calculated using the Fisher test and adjusted for multiple hypotheses. The combined score corresponds to multiplication of the log p-value and the z-score of the deviation from the expected rank of that term in random lists of genes. The top 15 functional enrichment terms for each cluster are ranked according to the adjusted p-value and displayed in a tabular format when the mouse hovers over a node in that cluster. Each cluster can be visualized separately by “Select by group” icon located at the top of the figure.

```
plot(res$subnet, edge_width = 8, node_size = 30, node_label_cex = 1)
```

In this case, the presence of subnetworks enriched for Gene Ontology terms like ‘mesenchymal-epithelial cell signaling’ and ‘EGFR downregulation’ confirm the importance of these pathways in this lung cancer model. We also see Steiner nodes such as CBL, which has been shown to be involved in several models of non-small cell lung cancers (Y.-H. C. Tan et al. 2010), like the H358 cell line. Therefore, we see that the PSCF algorithm points out proteins and pathways that are highly relevant to the system under study.

#### REFERENCES

Akhmedov, Murodzhon, Ivo Kwee, and Montemanni Roberto. 2016. “A Divide and Conquer Matheuristic Algorithm for the Prize-Collecting Steiner Tree Problem.” *Computers and Operations Research* 70: 18–25.

Akhmedov, Murodzhon, Alex LeNail, Francesco Bertoni, Ivo Kwee, Ernest Fraenkel, and Montemanni Roberto. 2017. “A Fast Prize-Collecting Steiner Forest Algorithm for Functional Analyses in Biological Networks.” *Lecture Notes in Computer Science*, 263–76.

Alexa, Adrian, and Jorg Rahnenfuhrer. 2009. “TopGO: Enrichment Analysis for Gene Ontology.” *R Package Version 2.28.0*.

Bailly-Bechet, Marc, Christian Borgs, Alfredo Braunstein, J Chayes, A Dagkessamanskaia, J-M François, and Riccardo Zecchina. 2011. “Finding Undetected Protein Associations in Cell Signaling by Belief Propagation.” *Proceedings of the National Academy of Sciences* 108 (2). National Acad Sciences: 882–87.

Chen, Edward Y, Christopher M Tan, Yan Kou, Qiaonan Duan, Zichen Wang, Gabriela Vaz Meirelles, Neil R Clark, and Avi Ma’ayan. 2013. “Enrichr: Interactive and Collaborative Html5 Gene List Enrichment Analysis Tool.” *BMC Bioinformatics* 14 (1). BioMed Central: 1.

Dittrich, Marcus T, Gunnar W Klau, Andreas Rosenwald, Thomas Dandekar, and Tobias Müller. 2008. “Identifying Functional Modules in Protein–protein Interaction Networks: An Integrated Exact Approach.” *Bioinformatics* 24 (13). Oxford Univ Press: i223–i231.

Szklarczyk, Damian, Andrea Franceschini, Michael Kuhn, Milan Simonovic, Alexander Roth, Pablo Minguez, Tobias Doerks, et al. 2011. “The String Database in 2011: Functional Interaction Networks of Proteins, Globally Integrated and Scored.” *Nucleic Acids Research* 39 (suppl 1). Oxford Univ Press: D561–D568.

Tan, Yi-Hung Carol, Soundararajan Krishnaswamy, Suvobroto Nandi, Rajani Kanteti, Sapana Vora, Kenan Onel, Rifat Hasina, et al. 2010. “CBL Is Frequently Altered in Lung Cancers: Its Relationship to Mutations in Met and Egfr Tyrosine Kinases.” *PloS One* 5 (1). Public Library of Science: e8972.

Thomson, Stuart, Filippo Petti, Izabela Sujka-Kwok, Peter Mercado, James Bean, Melissa Monaghan, Sean L Seymour, Gretchen M Argast, David M Epstein, and John D Haley. 2011. “A Systems View of Epithelial–mesenchymal Transition Signaling States.” *Clinical & Experimental Metastasis* 28 (2). Springer: 137–55.

Tuncbag, Nurcan, Sara JC Gosline, Amanda Kedaigle, Anthony R Soltis, Anthony Gitter, and Ernest Fraenkel. 2016. “Network-Based Interpretation of Diverse High-Throughput Datasets Through the Omics Integrator Software Package.” *PLoS Comput Biol* 12 (4). Public Library of Science: e1004879.
