# Supplementary material for: PCSF: An R-package for network-based interpretation of high-throughput data
Source: PLoS Comput Biol. 2017 Jul 31;13(7):e1005694. doi: 10.1371/journal.pcbi.1005694 (PMC5552342; doi:10.1371/journal.pcbi.1005694)
Supplement: S2 File — (PDF) [file pcbi.1005694.s002.pdf]

Package ‘PCSF’

July 12, 2017

Type Package

Title Network-based interpretation of highthroughput data

Version 0.99.0

Date 2017-02-01

Author Murodzhon Akhmedov, Amanda Kedaigle, Renan Escalante, Roberto Montemanni, Francesco Bertoni, Ernest Fraenkel, Ivo Kwee

Maintainer Murodzhon Akhmedov <murodzhon@idsia.ch>

Description The PCSF package performs an integrated analysis of highthroughput data using the interaction networks as a template, and interprets the biological landscape of interaction networks with respect to the data, which potentially leads to predictions of functional units. It also interactively visualize the resulting subnetwork with functional enrichment analysis.

License MIT + file LICENSE

Suggests knitr, rmarkdown

Depends R (>= 3.1.0), igraph, visNetwork,

Imports BH, httr, methods, org.Hs.eg.db, Rcpp, topGO

LinkingTo Rcpp, BH

RoxygenNote 6.0.1

NeedsCompilation yes

R topics documented:

construct_interactome 2
enrichment_analysis 2
PCSF 4
PCSF_rand 6
plot.PCSF 7
plot.PCSFe 8
STRING 9
Tgfb_phospho 10

Index **11**

construct_interactome *Construct an interaction network*

Description

Given a list of edges, construct_interactome generates an interaction network which is used as a template network to interpret the highthrougput data.

Usage

```
construct_interactome(ppi)
```

Arguments

ppi	A list of edges. A data.frame composed of three columns, where each row corresponds to an edge in which the first element is a head, the second element is a tail, and the last element represents the cost of the edge.

Value

An interaction network as **igraph** object.

Author(s)

Murodzhon Akhmedov

Examples

```
## Not run:
library("PCSF")
data("STRING")
ppi <- construct_interactome(STRING)
## End(Not run)
```

enrichment_analysis *Perform enrichment analysis on the subnetwork*

Description

enrichment_analysis performs functional enrichment analysis on the subnetwork obtained by the PCSF_rand, and returns an annotated subnetwork with top 15 functional enrichments and a list of tables with a complete enrichment analysis for each cluster.

Usage

```
enrichment_analysis(subnet, mode = NULL, gene_universe)
```

Arguments

subnet	A subnetwork provided by PCSF_rand, which is obtained by merging a multiple outputs of the PCSF with random noise added edge costs. An **igraph** object with edge cost and vertex prize attributes representing the total number of show ups throughout all runs.
mode	A binary variable to choose the method for enrichment analysis, where 0 is for EnrichR API and 1 is for **topGO** package.
gene_universe	A complete list of genes (vector of gene symbols) used as background in enrichment analysis by **topGO** package.

Details

An enrichment analysis of the final subnetwork obtained by multiple runs of the PCSF (with rando noise added edge costs) is performed for functional interpretation. The subnetwork is clustered using an edge betweenness clustering algorithm from the **igraph** package, and for each cluster functional enrichment is done by employing either EnrichR API (Chen *et al.*, 2013) or **topGO** (Alexa and Rahnenfuhrer, 2009) package that is specified by the user. Important to note that EnrichR API requires a working Internet connection to perform the enrichment. If the user does not specify which tool to use for enrichment analysis, the package employs EnrichR as a default if there is Internet connection, otherwise it uses **topGO**.

An interactive visualization of the final subnetwork is plotted, where the node sizes and edge widths are proportional to the frequency of show ups throughout total runs. Nodes are colored according to the cluster membership, and the top 15 functional enrichment terms are displayed in tabular format during the hover-over of the node in that cluster.

Value

A list composed of an interactive subnetwork and a table with enrichment analysis results. An interactive subnetwork annotated with enrichment analysis can be reached by $subnet. A full list of enrichment analysis for each cluster can be reached by $enrichment.

Author(s)

Murodzhon Akhmedov

References

Chen E.Y., Christopher M.T., Yan K., Qiaonan D., Zichen W., Gabriela V.M., Neil R.C., and Avi M. (2013) Enrichr: Interactive and Collaborative Html5 Gene List Enrichment Analysis Tool. *BMC Bioinformatics* 14 (1). BioMed Central: 1.

Alexa A. and Rahnenfuhrer J. (2009). topGO: Enrichment Analysis for Gene Ontology. R package version 2.28.0.

See Also

PCSF_rand, plot.PCSFe

Examples

```
## Not run:
library("PCSF")
data("STRING")
```

```
data("Tgfb_phospho")
terminals <- Tgfb_phospho
ppi <- construct_interactome(STRING)
subnet <- PCSF_rand(ppi, terminals, n = 10, r = 0.1, w = 2, b = 1, mu = 0.0005)
res <- enrichment_analysis(subnet)
res <- enrichment_analysis(subnet, mode=0)
## End(Not run)
## Not run:
library(topGO)
gene_universe <- V(ppi)$name
res <- enrichment_analysis(subnet, mode=1, gene_universe)
## End(Not run)
## Not run:
plot(res$subnet)
write.table(res$enrichment[[1]],file="cluster1_complete_enrichment.txt",
            append = FALSE, quote = FALSE, sep ="\t", row.names=FALSE)
## End(Not run)
```

PCSF *Prize-collecting Steiner Forest (PCSF)*

Description

PCSF returns a subnetwork obtained by solving the PCSF on the given interaction network.

Usage

```
PCSF(ppi, terminals, w = 2, b = 1, mu = 5e-04)
```

Arguments

ppi	An interaction network, an **igraph** object.
terminals	A list of terminal genes with prizes to be analyzed in the PCSF context. A named numeric vector, where terminal genes are named same as in the interaction network and numeric values correspond to the importance of the gene within the study.
w	A numeric value for tuning the number of trees in the output. A default value is 2.
b	A numeric value for tuning the node prizes. A default value is 1.
mu	A numeric value for a hub penalization. A default value is 0.0005.

Details

The PCSF is a well-know problem in graph theory. Given an undirected graph *G = (V, E)*, where the vertices are labeled with prizes p_v and the edges are labeled with costs $c_e > 0$, the goal is to identify a subnetwork *G' = (V', E')* with a forest structure. The target is to minimize the total edge costs in *E'*, the total node prizes left out of *V'*, and the number of trees in *G'*. This is equivalent to minimization of the following objective function:

$$F(G') = Minimize \sum_{e \in E'} c_e + \beta * \sum_{v \notin V'} p_v + \omega * k$$

where, *k* is the number of trees in the forest, and it is regulated by parameter ω. The parameter β is used to tune the prizes of nodes.

This optimization problem nicely maps onto the problem of finding differentially enriched subnetworks in the cell protein-protein interaction (PPI) network. The vertices of interaction network correspond to genes or proteins, and edges represent the interactions among them. We can assign prizes to vertices based on measurements of differential expression, copy number, or mutation, and costs to edges based on confidence scores for those intra-cellular interactions from experimental observation, yielding a proper input to the PCSF problem. Vertices that are assigned a prize are referred to *terminal* nodes, whereas the vertices which are not observed in patient data are not assigned a prize and are called *Steiner* nodes. After scoring the interactome, the PCSF is used to detect a relevant subnetwork (forest), which corresponds to a portion of the interactome, where many genes are highly correlated in terms of their functions and may regulate the differentially active biological process of interest. The PCSF aims to identify neighborhoods in interaction networks potentially belonging to the key dysregulated pathways of a disease. In order to avoid a bias towards the hub nodes of PPI networks to appear in solution of PCSF, we penalize the prizes of *Steiner* nodes according to their degree distribution in PPI, and it is regulated by parameter μ:

$$p'_v = p_v - \mu * degree(v)$$

The parameter μ also affects the total number of *Steiner* nodes in the solution. Higher the value of μ smaller the number of *Steiners* in the subnetwork, and vice-versa. Based on our previous analysis the recommended range of μ for biological networks is between 1e-4 and 5e-2, and users can choose the values resulting subnetworks with vertex sets that have desirable *Steiner/terminal* node ratio and average *Steiner/terminal* in-degree ratio in the template interaction network.

Value

The final subnetwork obtained by the PCSF. It return an **igraph** object with the node prize and edge cost attributes.

Author(s)

Murodzhon Akhmedov

References

Akhmedov M., LeNail A., Bertoni F., Kwee I., Fraenkel E., and Montemanni R. (2017) A Fast Prize-Collecting Steiner Forest Algorithm for Functional Analyses in Biological Networks. *Lecture Notes in Computer Science*, to appear.

See Also

PCSF_rand, plot.PCSF

Examples

```
## Not run:
library("PCSF")
data("STRING")
data("Tgfb_phospho")
terminals <- Tgfb_phospho
ppi <- construct_interactome(STRING)
subnet <- PCSF(ppi, terminals, w = 2, b = 1, mu = 0.0005)
## End(Not run)
```

PCSF_rand *Prize-collecting Steiner Forest (PCSF) with randomized edge costs*

Description

PCSF_rand returns a union of subnetworks obtained by solving the PCSF on the given interaction network by adding a random noise to edge costs each time.

Usage

```
PCSF_rand(ppi, terminals, n = 10, r = 0.1, w = 2, b = 1, mu = 5e-04)
```

Arguments

ppi	An interaction network as an **igraph** object.
terminals	A list of terminal genes with prizes to be analyzed in the PCSF context. A named numeric vector, where terminal genes are named same as in the interaction network and numeric values correspond to the importance of the gene within the study.
n	An integer value to determine the number of runs with random noise added edge costs. A default value is 10.
r	A numeric value to determine additional random noise to edge costs. A random noise upto r percent of the edge cost is added to each edge. A default value is 0.1
w	A numeric value for tuning the number of trees in the output. A default value is 2.
b	A numeric value for tuning the node prizes. A default value is 1.
mu	A numeric value for a hub penalization. A default value is 0.0005.

Details

In order to increase the robustness of the resulting structure, it is recommended to solve the PCSF several times on the same network while adding some noise to the edge costs each time, and combine all results in a final subnetwork. The union of all outputs may explain the underlying biology better.

Value

The final subnetwork obtained by taking the union of the PCSF outputs generated by adding a random noise to edge costs each time. It returns an **igraph** object with the node prize and edge cost attributes representing the total number of show ups throughout all runs.

Author(s)

Murodzhon Akhmedov

References

Akhmedov M., LeNail A., Bertoni F., Kwee I., Fraenkel E., and Montemanni R. (2017) A Fast Prize-Collecting Steiner Forest Algorithm for Functional Analyses in Biological Networks. *Lecture Notes in Computer Science*, to appear.

See Also

PCSF, plot.PCSFe

Examples

```
## Not run:
library("PCSF")
data("STRING")
data("Tgfb_phospho")
terminals <- Tgfb_phospho
ppi <- construct_interactome(STRING)
subnet <- PCSF_rand(ppi, terminals, n = 10, r =0.1, w = 2, b = 2, mu = 0.0005)
## End(Not run)
```

---

plot.PCSF *Plot an interactive subnetwork*

Description

plot.PCSF plots an interactive figure of the subnetwork obrained by the PCSF method.

Usage

```
## S3 method for class 'PCSF'
plot(x, style = 0, edge_width = 5, node_size = 40,
  node_label_cex = 30, Steiner_node_color = "lightblue",
  Terminal_node_color = "lightgreen", ...)
```

Arguments

x	A subnetwork obtained by the PCSF method. It is a "PCSF" object derived from **igraph** class and it has the edge cost and vertex prize attributes.
style	A boolean value to determine the visualization style of the network, where 0 plots the static network and 1 plots the dynamic network. The default valu is 0.
edge_width	A numeric value to emphasize the maximum edge width. A default value is 5. This value must be greater than 1.
node_size	A numeric value to emphasize the maximum node size. A default value is 40. This value must be greater than 10.
node_label_cex	A numeric value to set the node label size. A default value is 30.
Steiner_node_color	A string to set the color of Steiner nodes. A default value is "lightblue".
Terminal_node_color	A string to set the color of terminal nodes. A default value is "lightgreen".
...	Ignored.

Details

This function plots an interactive subnetwork obtained by the PCSF and PCSF_rand. The node sizes and edge widths are respectively proportional to the node prizes and edge costs while plotting the subnetwork from PCSF. In contrast, the node sizes and edge widths are proportional to the total number of abondance in randomized runs while plotting the subnetwork from PCSF_rand. The node names are displayed during the hover-over.

Author(s)

Murodzhon Akhmedov

See Also

PCSF, plot.PCSFe

Examples

```
## Not run:
library("PCSF")
data("STRING")
data("Tgfb_phospho")
terminals <- Tgfb_phospho
ppi <- construct_interactome(STRING)
subnet <- PCSF(ppi, terminals, w = 2, b = 1, mu = 0.0005)
plot(subnet)
## End(Not run)
```

---

plot.PCSFe — *Plot an interactive subnetwork with functional enrichment analysis*

Description

plot.PCSFe plots an interactive figure of the subnetwork to display the functionla enrichment analysis, which is obtained by employing enrichment_analysis on the subnetwork.

Usage

```
## S3 method for class 'PCSFe'
plot(x, edge_width = 5, node_size = 30,
  node_label_cex = 1, ...)
```

Arguments

x	An output subnetwork provided by the enrichment_analysis. It is "PCSFe" object derived from an **igraph** class, and it has the edge cost and vertex prize attributes.
edge_width	A numeric value to emphasize the maximum edge width. A default value is 5. This value must be greater than 1.
node_size	A numeric value to emphasize the maximum node size. A default value is 30. This value must be greater than 10.
node_label_cex	A numeric value to set the node label size. A default value is 1.
...	Ignored.

Details

An enrichment analysis of the final subnetwork obtained by multiple runs of the PCSF (with random noise added edge costs) is performed by using `enrichment_analysis`. The subnetwork is clustered using an edge betweenness clustering algorithm from the **igraph** package, and for each cluster functional enrichment is done by employing the ENRICHR API (Chen *et al.*, 2013). An interactive visualization of the final subnetwork is plotted, where the node sizes and edge widths are proportional to the frequency of show ups in total randomised runs. Nodes are colored according to the cluster membership, and the top 15 functional enrichment terms are displayed in tabular format during the hover-over of the node in that cluster. A specific cluster can be displayed separately in the figure by selecting from the icon list at the top left side of the figure.

Author(s)

Murodzhon Akhmedov

References

Chen E.Y., Christopher M.T., Yan K., Qiaonan D., Zichen W., Gabriela V.M., Neil R.C., and Avi M. (2013) Enrichr: Interactive and Collaborative Html5 Gene List Enrichment Analysis Tool. *BMC Bioinformatics* 14 (1). BioMed Central: 1.

See Also

`enrichment_analysis`, `PCSF_rand`, `plot.PCSF`

Examples

```
## Not run:
library("PCSF")
data("STRING")
data("Tgfb_phospho")
terminals <- Tgfb_phospho
ppi <- construct_interactome(STRING)
subnet <- PCSF_rand(ppi, terminals, n = 10, r = 0.1, w = 2, b = 1, mu = 0.0005)
res <- enrichment_analysis(subnet)
plot(res$subnet)
## End(Not run)
```

`STRING` *Protein-protein interaction network data*

Description

An interactome data set in which the nodes are named with gene symbols

Usage

```
STRING
```

Format

A data frame with three variables, where each row corresponds to an edge in which the first element is a head, the second element is a tail, and the last element represents the cost of the edge.

Source

iref_mitab_miscore_2013_08_12_interactome.txt https://github.com/fraenkel-lab/OmicsIntegrator/tree/master/data

---

Tgfb_phospho *Phosphoproteomic data*

Description

This dataset contains differential phosphoproteomic data derived from H358 cells, a model of lung cancer, that were stimulated with TGF-b.

Usage

```
Tgfb_phospho
```

Format

A named numeric vector, where terminal genes are named same as in the interaction network and numeric values correspond to the importance of the gene within the study.

Source

Tgfb_phos.txt https://github.com/fraenkel-lab/OmicsIntegrator/tree/master/example/a549

Index

∗Topic **data**
STRING, 9
Tgfb_phospho, 10

construct_interactome, 2

enrichment_analysis, 2, *9*

PCSF, 4, *7*, *8*
PCSF_rand, *2*, *3*, *5*, 6, *8*, *9*
plot.PCSF, *5*, 7, *9*
plot.PCSFe, *3*, *7*, *8*, 8

STRING, 9

Tgfb_phospho, 10
